# Immune Regulation and the Role of Extracellular Vesicles in Non-Small Cell Lung Cancer: Biological Mechanisms and Therapeutic Perspectives

**DOI:** 10.3390/ph19071023

**Published:** 2026-06-30

**Authors:** Nicole Ferrario, Orazio Fortunato, Patrizia Ghidotti

**Affiliations:** Unit of Epigenomics and Biomarkers of Solid Tumors, Fondazione IRCCS Istituto Nazionale dei Tumori, 20133 Milan, Italy; nicole.ferrario@istitutotumori.mi.it (N.F.); patrizia.ghidotti@istitutotumori.mi.it (P.G.)

**Keywords:** extracellular vesicles, lung cancer, immune system

## Abstract

Lung cancer remains one of the leading causes of cancer-related mortality worldwide, with non-small cell lung cancer (NSCLC) representing the most common subtype. Despite major advances in immunotherapy, only a subset of patients benefits from current treatments, highlighting the need to better understand the tumor immune microenvironment (TIME) and the mechanisms underlying immune escape. In this context, extracellular vesicles (EVs) have emerged as key mediators of intercellular communication in lung cancer. This review summarizes the current knowledge on the role of EVs in NSCLC progression and immune regulation. We discuss how EVs contribute to primary tumor growth, dissemination, and pre-metastatic niche formation through the transfer of proteins, metabolites and nucleic acids. Particular attention is given to EV-mediated modulation of immune cells, highlighting their role in both immune suppression and immune activation. Furthermore, we provide an overview of the emerging therapeutic applications of EVs in lung cancer, including their use as drug-delivery systems and immunotherapeutic platforms.

## 1. Lung Cancer

Lung cancer (LC) is among the most common malignancies worldwide and remains the leading cause of cancer-related mortality [1]. According to the Global cancer statistics, it has the highest incidence and mortality rates in men, while in women, it ranks second for both incidence and mortality [2]. Despite advances in screening programs and the development of innovative diagnostic and therapeutic techniques aimed at improving prognosis, LC continues to represent a major clinical and social challenge, as well as a significant economic burden for both healthcare systems and patients [1,3].

LC is a multifactorial disease influenced by various environmental (e.g., infections, ionizing radiation, occupational exposures, air pollution) and genetic risk factors (e.g., genetic polymorphisms) [4]. However, tobacco smoking remains the primary cause of its development [5].

Clinically, LC is classified into two main types: non-small cell lung cancer (NSCLC), which accounts for the majority of cases and is associated with a poor prognosis, with a 5-year survival rate of approximately 20% [6], and small-cell lung cancer (SCLC) [5,7]. NSCLC is further subdivided by the World Health Organization (WHO) into three main histological subtypes: adenocarcinoma (40%), squamous cell carcinoma (25–30%), and large cell carcinoma (5–10%) [5]. Moreover, LC could also be classified into 4 stages, from stage I to stage IV, based on the dimension of the primary tumor, involvement of lymph nodes, and metastatic spread. In particular, stage IV describes a tumor characterized by a metastatic disease with spread of tumor cells in nearby tissues, nodules and distant organs [8].

Primarily due to the presence of quite specific disease manifestations such as dyspnea, persistent cough, hemoptysis, chest pain and weight loss, the diagnosis of LC is often made when the disease has already reached an advanced stage [9]. The delay in the diagnosis further complicates both the clinical management and the therapeutic approach of NSCLC patients. Indeed, while early-stage LC can be effectively treated by surgery [10,11], treatment options for patients with advanced disease are considerably less effective [12]. Nevertheless, the advent of novel therapeutic strategies such as immunotherapy and/or a combination of chemotherapy plus immunotherapy led to significant improvements in clinical outcomes, in terms of overall survival (OS) and progression-free survival (PFS) in metastatic NSCLC patients [13,14,15].

Notably, immunotherapy takes advantage of the host immune system to achieve a clinical response [16,17], and among the most relevant immunotherapeutic agents are immune checkpoint blockers (ICBs), monoclonal antibodies that target immune checkpoint molecules expressed on tumor-infiltrating lymphocytes, cancer cells, and other components of the tumor microenvironment (TME) [18]. These immune checkpoint receptors can deliver either activating signals, such as CD28 and ICOS, or inhibitory signals, like Cytotoxic T-lymphocyte-associated protein 4 (CTLA-4), programmed cell death protein 1 (PD-1) and programmed death-ligand 1 (PD-L1), thereby shaping T cell activity [17,19]. In particular, PD-1 and PDL-1, which are respectively expressed on activated lymphocytes and on tumor cells, in physiological conditions suppress cytotoxic T cell responses. Likewise, CTLA-4, another inhibitory checkpoint receptor expressed on T cells, negatively regulates T cell priming and activation [20]. In the cancer context, however, tumor cells and non-malignant cells of the TME can up-regulate the expression of inhibitory immune checkpoint ligands as a mechanism of immune escape, ultimately impairing T cell function and promoting tumor progression [21]. Thus, ICBs restore an effective anti-tumor immunity by blocking inhibitory checkpoint signals and reactivating T cell response [22].

Several clinical trials have demonstrated the therapeutic benefit of ICBs in NSCLC. For instance, the KEYNOTE-024 trial showed that Pembrolizumab significantly improved the five-year OS rate compared with platinum-based chemotherapy in metastatic NSCLC patients with high PD-L1 expression (>50%), from 16.3% to 31.9% [23,24]. Moreover, besides single-agent immunotherapy, combination approaches involving multiple immunotherapeutic agents or the integration of chemotherapy with immunotherapy have also emerged as promising treatment strategies for NSCLC management [25,26]. Indeed, the Checkmate 227 trial showed that patients treated with a combination of Ipilimumab, a CTLA-4 inhibitor, plus Nivolumab, an anti-PD-1 monoclonal antibody, increased 5-year survival rates compared to chemotherapy alone. Indeed, patients who were administered the combination of chemotherapy plus immunotherapy estimated an OS rate of 24% compared to 14% of chemotherapy alone [27]. Similarly, a combination of Ipilimumab and chemotherapy highlighted benefits in terms of PFS compared to Ipilimumab alone in advanced NSCLC patients [28].

However, despite the promising advent of those new therapeutic strategies, only a small subset of patients takes advantage of immunotherapy, and resistance still remains a major challenge, even when combined with chemotherapy [29,30,31]. This highlights the critical need to better understand the role of the immune system within the lung TME and its interactions with tumor cells to unveil possible solutions to those clinical issues. Among the factors that are involved in tumor-immune system interaction, there are extracellular vesicles (EVs), membrane-limited particles strongly involved in all the steps of cancer [32]. This review aims to provide a clear overview of what current literature reports on the involvement of EVs in LC and, in particular, how these particles influence immune system functions during cancer, with strong implications for response to therapy and future therapeutic opportunities.

## 2. The Relevance of Immune System-Tumor Interaction in Cancer Progression

The immune system plays a central role in tumorigenesis, as it can both eliminate cancer cells and support tumor growth [33]. Indeed, cancer cells and the immune system interact with each other during cancer development through a process called “immunoediting” [34], which counts three main phases: elimination, equilibrium and escape [34,35]. Briefly, the elimination phase involves innate and adaptive immunity, which cooperate to identify and eradicate cancer cells [33]. Tumor cells that evade the elimination phase enter the equilibrium phase, in which they remain dormant, but their immunogenicity is still under pressure from the adaptive immunity [21,33]. Finally, the adoption of immunoevasion mechanisms (e.g., defective antigen presentation, loss of major histocompatibility complex molecules (MHC)) and immunosuppressive mechanisms (e.g., immune checkpoint inhibitory molecule expression, recruitment of pro-tumoral immune cell populations) within the tumor can ultimately lead them to escape the immune surveillance and restart tumor growth [33].

The constitution of an immunosuppressive microenvironment represents a key event for a successful immune escape orchestrated by cancer cells [35]. Key helpers of tumor growth in this context include regulatory T (Treg) cells, myeloid-derived suppressor cells (MDSCs), and tumor-associated macrophages (TAMs). In particular, Tregs contribute to suppressing tumor-specific T cells and Natural Killer (NK) cells, while MDSCs promote Treg expansion and inhibit T cell proliferation. TAMs and stromal cells, instead, promote the formation of an immunosuppressive microenvironment mainly through the release of IL-10 and TGF-β cytokines [36,37] as well as through the activation of Treg populations [33,35].

Tumor immune composition is also fundamental when considering immunotherapy and response to treatment. Indeed, three main tumor immune microenvironments can be distinguished: immune-infiltrated, immune-excluded and immune-deserted [38]. Immune-infiltrated tumors, or “hot” tumors, are characterized by abundant PD1^+^ cytotoxic CD8^+^ T lymphocytes (CTLs) and high tumor mutational burden (TMB), making them more responsive to ICBs. Conversely, immune-excluded tumors show poor immune infiltration [39]. Immune-deserted, or “cold” tumors, have limited T cell infiltration and are enriched in immunosuppressive cells and exhibit low PD-L1 expression as well as low TMB, thus contributing to their resistance to ICB therapy [40].

In the setting of LC, numerous studies have highlighted that despite the presence of tumor-infiltrating lymphocytes, the majority of LC cases show a cold or immune-excluded microenvironment [41]. Nevertheless, among them, we have to consider variability since the immune microenvironment could vary depending on the smoking history [42] and the TMB, which can influence immunogenicity and immune system response in NSCLC [43,44].

## 3. Tumor Immune Microenvironment in Lung Cancer

**Figure 1:**
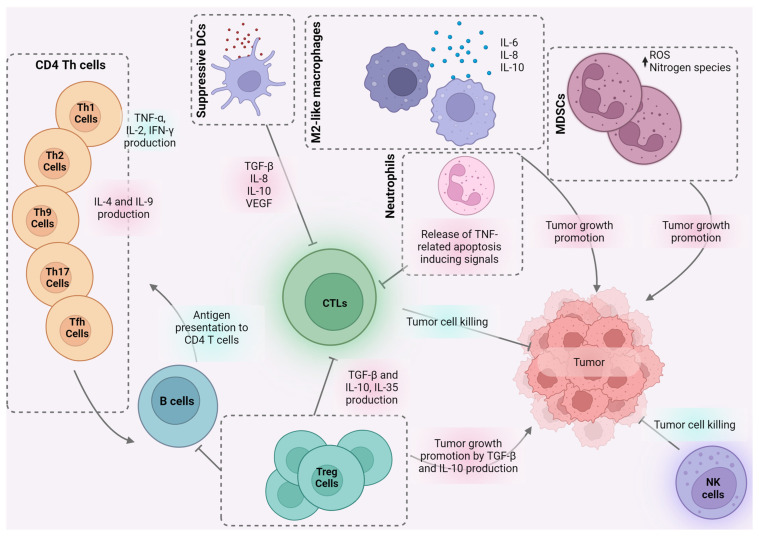
Immune cells involved in cancer. The TIME comprehends both innate and adaptive immune cells, and their behavior is strongly influenced by tumor cells. At the same time, the balance between pro-tumoral immune populations (e.g., M2 macrophages, MDSCs, and neutrophils) and tumor-suppressive populations (e.g., CTLs, Th cells) strongly affects the progression or the suppression of cancer. Created in BioRender. Ghidotti, P. (2026) https://BioRender.com/9jytsic (accessed on 19 June 2026).

The lung tumor immune microenvironment (TIME) has been extensively characterized, and many of the key mechanisms regulating lung immune responses are shared across different tumor types (Table 1). During cancer promotion, different immune cell populations take part in the promotion or inhibition of the growth and progression of cancer cells (Figure 1).

In addition to shared immune regulatory mechanisms, LC displays several unique features that might distinguish its immune landscape from others. One major determinant is smoking status, which influences both the genomic feature and immune composition of lung tumors [55]. Nicotine reduced granzyme B expression in CD8^+^ T cells, and smoking-induced miR-629-5p epigenetic modifications decrease IL-2 receptor expression, thereby attenuating CD8^+^ T-cell activation [56]. Tobacco exposure in lung cancer patients further impairs dendritic cell function by reducing their antigen-presenting capacity [57], while promoting a shift of tumor-associated macrophages (TAMs) from a pro-inflammatory M1 phenotype toward an immunosuppressive M2 phenotype [58].

Beyond its direct effects on immune cells, smoking contributes to the accumulation of genomic alterations, and these molecular differences can impact antitumor immunity [59]. Indeed, NSCLC harboring EGFR mutations exhibits a distinct TME in which EGFR activation promotes PD-L1 expression on tumor cells, leading to T-cell apoptosis and immune evasion [60]. Similarly, KRAS^G12V^ overexpression induces PD-L1 upregulation through epithelial-to-mesenchymal transition (EMT), thereby facilitating immune escape [61]. Consistent with the notion that oncogenic drivers shape immune composition, Hartner and colleagues demonstrated that lung tumors harboring EGFR, PIK3CA, or unknown driver alterations exhibited a higher lymphoid-to-myeloid cell ratio, whereas KRAS-, BRAF-, and MET-driven tumors displayed the opposite pattern [62].

In addition to specific oncogenic driver alterations, the overall number of somatic mutations within a tumor, known as tumor mutational burden (TMB), may also influence antitumor immune responses [63]. Lung adenocarcinomas with high TMB are thought to generate a broader amount of neoantigens, thereby enhancing immune recognition and increasing responsiveness to immune checkpoint inhibitors [64,65].

Another distinctive feature of lung cancer is the heterogeneity of the immune landscape across histological subtypes. Transcriptomic analyses of the TIME have shown that lung adenocarcinoma (LUAD) generally displays greater immune cell infiltration than lung squamous cell carcinoma (LUSC), particularly by CD8^+^ T cells. Moreover, adenocarcinomas exhibit a higher neoantigen load, CD8^+^ T-cell infiltration, and greater T-cell receptor (TCR) clonal expansion, all indicative of a more active immune microenvironment. Furthermore, macrophages, neutrophils and mast cells were also found to be more present in adenocarcinoma compared with squamous cell carcinoma [66]. Similarly, Wang and colleagues have also found that LUAD contain higher proportions of effector and activated immune cells and lower frequencies of Tregs than LUSC, further supporting the existence of subtype-specific immune ecosystems within lung cancer [67].

Finally, LUAD have been shown to be carriers of tertiary lymphoid structures (TLSs), lymphoid formations with organized T and B lymphocyte colonies, that have been positively correlated with the number of mutations/neoantigen load, in tumors with high mutation or neoantigen load, therefore rendering them strong predictors of survival and immunotherapy efficacy [68].

Despite the well-established importance of the cellular component in regulating immunity in the tumor context, soluble mediators also play a fundamental role in immunoregulation. Indeed, cytokines, chemokines, and soluble immune checkpoint factors have been demonstrated to be powerful contributors in this setting. In addition, alongside these molecules, EVs have recently emerged as key mediators of intercellular communication within the tumor immune microenvironment and in the transmission of immunomodulatory signals, highlighting their relevance in tumor immunity and their promise in the development of novel therapeutic strategies [69].

## 4. Extracellular Vesicles

EVs are lipid bilayer membrane-delimited, nano- to micro-sized particles that appear to be released by all cell types and cannot replicate on their own [70]. The term EV includes a variety of vesicles which are different depending on the pathway of origin, dimension and/or composition [71]. As interest in EVs has grown rapidly, researchers have increasingly focused on understanding their biological features, including their protein [72,73] and lipid composition [74]. Parallelly, many studies have shown that EVs are involved in both physiological and pathological processes, particularly highlighting their roles in immune regulation [75] and cancer [76]. Despite the huge amount of experimental evidence that documents the involvement of EVs in cancers, it is also worth noting that these evidences often come from in vitro experiments. In these conditions, cell lines are exposed to a huge number of vesicles that, with low probability, represent the real amount of EVs to which cancer cells and cells of the microenvironment are exposed [70,77]. For this reason, it is often difficult to correlate what happens in in vitro models to what goes on in vivo, and certain tumor-promoting or tumor-inhibiting mechanisms may not be so relevant and fade into the background [78].

EVs are now widely recognized as important mediators of intercellular communication, as they can transfer bioactive molecules, which include proteins, metabolites, mRNAs, and microRNAs, to recipient cells, thereby influencing their function [79]. The transfer of these biomolecules relies on the capacity of EVs to act as autocrine, paracrine, and endocrine messengers. In the context of cancer, the autocrine activity of EVs is particularly relevant since it allows cancer cells to self-sustain their aggressiveness and proliferation in early stages of tumorigenesis [80,81]. Instead, paracrine and endocrine properties of EVs are pivotal for the corruption of the tumor microenvironment and for the constitution of distant metastases, respectively [82].

Consistent with this role, EVs are broadly implicated in cancer initiation, progression and metastasis [83], and growing evidence suggests that circulating EVs may also systemically play a relevant role in immunomodulation by suppressing antitumor immunity [84]. For instance, they prevent CD8^+^ T cell proliferation and functionality, as they express on their surface inhibitory checkpoint ligands such as PD-L1 or CTLA-4 [85]. In particular, to exert their immunoregulatory function, EV-associated PD-L1 binds to PD-1 protein expressed on immune cells and dampens their activity [86]. Beyond immune checkpoint receptors, EVs can also display other transmembrane proteins involved in immunomodulation, including adhesion and signaling receptors (e.g., ICAM-1, integrins), antigen-presenting molecules such as MHC class I and II, or glycoproteins [87]. Integrins and adhesion molecules, for instance, facilitate EV interaction with recipient cells, promoting vesicle uptake and activation of intracellular signaling pathways [88]. MHC class I- and class II-bearing EVs, instead, are released by antigen-presenting cells (APCs) and may directly stimulate CD8^+^ and CD4^+^ T cells, respectively, thereby influencing adaptive immune responses [89].

In addition to proteins, EVs are also enriched in lipids that largely reflect the composition of their original cells, including sphingomyelin, cholesterol, ganglioside GM3, saturated lipids, phosphatidylserine, and ceramides [90]. Emerging evidence indicates that the EV lipidome is fundamental in immunomodulation, since, for example, specific lipids like sphingosine were associated with suppression of T cell activity, thereby contributing to the establishment of a pro-tumorigenic microenvironment [91].

Nucleic acids also represent an important component of EV cargo. Indeed, EVs can contain several forms of DNA, including single-stranded DNA, double-stranded DNA, genomic DNA, mitochondrial DNA, and reverse-transcribed complementary DNA, although their specific function is today still controversial [92]. In contrast, EVs are widely recognized as carriers of both coding RNAs, such as mRNAs, and non-coding RNAs, including long non-coding RNAs, microRNAs (miRNAs), and circular RNAs [93]. Among them, miRNAs were extensively studied and associated with a role in the regulation of immune cell signaling in LC [94]. For instance, in this context, microRNA-3127-5p has been shown to promote immune escape in LC cells through the induction of PD-L1 [95]. Similarly, circulating miR-320a, transported by polymorphonuclear neutrophil-derived EVs in high-risk heavy smokers, has been shown to induce a pro-tumorigenic M2-like phenotype in macrophages through STAT4 signaling [90]. Collectively, these findings highlight that EVs serve as complex carriers of proteins, lipids, and nucleic acids, with non-coding RNAs playing a particularly pivotal role in modulating tumor progression and immune responses. Through their ability to modulate both cancer cell behavior and immune cell function, EVs emerge as central mediators of tumor progression and immune regulation, as well as promising candidates for biomarker discovery and therapeutic intervention.

### 4.1. EVs in Lung Cancer Promotion

The involvement of EVs in all the steps of cancer establishment and progression has been largely documented [96,97]. Indeed, EVs released by cancer cells influence cancer cell behavior and those of other cells within the TME in order to foster cancer progression (Table 2).

#### 4.1.1. Primary Tumor Establishment and Growth

In different malignant cell types, an increased release of EVs has been described [107,113,114]. Concomitantly, these studies also reported that the inhibition of EV release reduces the malignant potential of the investigated tumor cell lines, suggesting a key role of EVs for cancer establishment and progression [97]. Increased EV release in the circulation seems to be relevant also in LC, since Choi et al. reported that LC patients showed higher circulating EV levels compared to healthy controls [115].

In NSCLC, the mutational state of parental cells also influences the properties of the released EVs, such as protein composition and miRNA cargo sorting. Indeed, EGFR mutation has been detected in circulating EVs derived from NSCLC patients [116,117] and LC cell lines [80], underlying the possibility of using EVs as a tool for the detection of common gene mutations in NSCLC. Moreover, EVs bearing mutated EGFR also increased cell proliferation, migration and invasion in recipient LC cell lines [80]. In our lab, we reported that different NSCLC cell line-derived EVs carry the oncoprotein c-Myc, which in turn influences the loading of miR-19b and miR-92a inside EVs [81]. When internalized by normal bronchial epithelial cells, c-Myc^+^ EVs induce the aberrant proliferation of recipient cells by targeting the TGF-β signaling pathway [81].

The acquisition of malignant traits by normal epithelial cells or the maintenance of aggressive properties by LC cells is also mediated by other molecules found in cancer-derived EVs. Indeed, Hasan et al. reported that RNA molecules sorted within NSCLC cell line-derived EVs induce the acquisition of aggressive properties in normal bronchial cell lines [98]. Instead, another study reported that α-SMA secreted via EVs and transferred to parental cells increases proliferation and inhibits apoptosis in parental cells, thus proposing an autocrine effect of EVs [99].

In addition to promoting local progression, EVs likewise support the dissemination and metastasis of the primary tumor [97]. During dissemination, cancer cells usually induce vascular permeability and angiogenesis in order to increase their chances of entering the bloodstream [118]. In different malignancies, it has been described that EVs shed by tumor cells carry different angiogenic molecules, such as Angiopoietin2 [119] in hepatocellular carcinoma-derived EVs, and E-cadherin in ovarian carcinoma-derived EVs [120]. In NSCLC, both proteins and miRNAs carried by EVs allow the formation of new vessels. A key mechanism to induce angiogenesis in LC seems to be mediated by the targeting of the TGF-β pathway [100,101]. Indeed, Li and colleagues reported that LRG1 transported by NSCLC cell line-derived EVs induced angiogenesis in human umbilical endothelial cells by targeting TFG-β [100]. Inhibition of the same pathway was described by miR-142-3p carried by LC cell line-derived EVs [101]. Indeed, miR-142-3p^+^ EVs by directly targeting TGF-β in endothelial cells significantly increase the formation of new vessels, which in turn support tumor cells [101]. Vascular permeability and angiogenesis are also induced by miR-23 shed via EVs by tumor cells exposed to hypoxic conditions. miR-23 inhibits PHD1 and 2 in endothelial cells, hence causing the accumulation of HIF-1α in endothelial cells that, in turn, enhances angiogenesis [102]. Furthermore, miR-23 targets ZO-1, with a consequent increase in vascular permeability [102]. Eventually, Wang and colleagues reported that EVs released by NSCLC cells promote angiogenesis in endothelial cells by interfering with YAP signaling, although without clear identification of the EV-associated components responsible for the described effect [103].

#### 4.1.2. Metastatic Spread

Once disseminated, cancer cells colonize distant organs and establish cancer metastasis [118]. Distant organ spreading is determined by intrinsic properties of cancer cells, but it is also influenced by many other systemic factors (e.g., release of EVs, cytokines) [97,118]. Successful metastatic establishment is strongly conditioned by the constitution of the so-called pre-metastatic niche (PMN). PMN constitution is intended as the process by which primary tumors, by exploiting different strategies, induce the re-organization of the microenvironment of distant organs by influencing matrix composition, stromal cells and immune cells behavior in order to create a favorable soil for metastasis establishment [121]. In this context, EVs are actively released by tumor cells, and they represent key players to achieve PMN constitution [113,121]. Indeed, Gou and colleagues showed that the modulation of EV release by inhibiting RAB27A expression, a central protein for EV biogenesis, reduces the metastatic potential of melanoma cell lines in vivo [122]. Instead, Peinado et al. underlined that highly metastatic melanoma cell lines produce EVs with a peculiar protein composition that, in turn, educates bone marrow to promote metastasis [113]. Moreover, in the past years, it has been highlighted that EVs also influence metastasis organotropisms based on the presence of specific integrin profiles on their surface [104]. Indeed, α6β4 and α6β1 were associated with lung metastasis, whereas integrin αvβ5 was associated with liver metastasis [104].

Extracellular matrix remodeling. One of the pivotal steps for PMN establishment is the remodeling of extracellular matrix (ECM), which mainly involves fibroblast activity [121,123]. ECM remodeling is intended as the modification of the already deposited ECM, the disruption of it, and the deposition of new components in order to create the best soil for the seeding of metastatic cells [123]. Different mechanisms of ECM remodeling are involved, considering the site of PMN constitution. Indeed, in the liver PMN of LC-bearing mice, Hsu and colleagues showed that miR-92a^+^ EVs released by monocytic myeloid-derived suppressor cells (M-MDSCs) and polymorphonuclear-MDSCs (PMN-MDSCs) influence the behavior of hepatic stellate cells [105]. Once internalized, miR-92a^+^ EVs induce the deposition of new ECM by hepatic stellate cells, which, in turn, promote the recruitment of MDSCs to the liver and the migration and invasion of cancer cells into the liver PMN [105]. Mechanistically, ECM deposition by hepatic stellate cells is induced by the targeting of SMAD7 by miR-92a and the consequent unleashing of TGF-β signaling. Interestingly, circulating miR-92a^+^ EVs derived from LC patients similarly induce the activation and ECM deposition in hepatic stellate cells, highlighting that miR-92a^+^ EVs may represent a crucial mediator of liver PMN formation also in patients [105]. Hepatic stellate cells also produce a higher quantity of fibronectin in response to TGF-β release by Kupffer cells in the context of pancreatic ductal adenocarcinoma [124]. Also in this case, fibronectin deposition supports the recruitment of MDSCs within the liver PMN [124]. Eventually, EVs derived from LC and melanoma cell lines mutated for p53 induced the production of a less organized ECM by fibroblast, which also favors the migration and invasion of cancer cells in the context of a lung PMN model [106].

Immunosuppression. Successful PMN establishment is also based on the constitution of an immunosuppressive microenvironment, starting from the recruitment of MDSCs, neutrophils, and other bone marrow-derived progenitor cells [121,123]. For the constitution of a lung PMN, different immunosuppressive mechanisms mediated by EVs have been described. In a model of breast cancer, the secretion of EVs is pivotal for the recruitment of neutrophils to the lung [107]. Indeed, inhibition of EV secretion via RAB27A silencing prevents primary tumor growth and lung dissemination in mice [107]. Moreover, Wen and colleagues also described that breast cancer-derived EVs accumulate in the lung and liver and are taken up by bone marrow-derived CD45^+^ cells [108]. Long-term injection of mice with breast cancer EVs induces the accumulation of MDSCs in the lung and liver, but also directly suppresses T cell proliferation and NK cytotoxicity, thereby favoring metastasis constitution [108]. Immune suppression can also be achieved via other stromal components of the niche. Indeed, in response to tumor-derived EVs with a tropism to the lungs, resident fibroblasts start to produce S100 that, in turn, induces the recruitment of DCs, macrophages, and neutrophils [104]. Moreover, fibroblast activity is also affected by miR-3473b^+^ EVs derived from Lewis lung carcinoma [109]. Indeed, EV uptake induces the production of pro-inflammatory cytokines by fibroblasts, thus participating in the constitution of an inflammatory niche that promotes lung invasion by cancer cells [109].

Vascular leakiness. PMN constitution foresees also altering the vascular permeability of endothelial cells in order to favor the migration of disseminating tumor cells into the metastatic site [121,123]. In metastatic NSCLC patients, Ma et al. described higher levels of miR-3157-3p^+^ circulating EVs than in non-metastatic patients [110]. Interestingly, when transferred to endothelial cells, miR-3157-3p^+^ EVs derived from tumor cells induced vascular leakiness and angiogenesis by targeting TIMP/KLF2, with the consequent increase in expression of MMP2, MMP9, VEGF and Occludin [110]. Eventually, in vivo transfer of these EVs increases lung metastatic nodule formation. Alteration of tight junction proteins to achieve vascular leakiness has also been described in other types of cancers, but mediated by other miRNAs [111,112,125], underlining the importance of endothelial alteration in PMN formation.

### 4.2. EV-Mediated Immune Regulation in Lung Cancer

**Figure 2:**
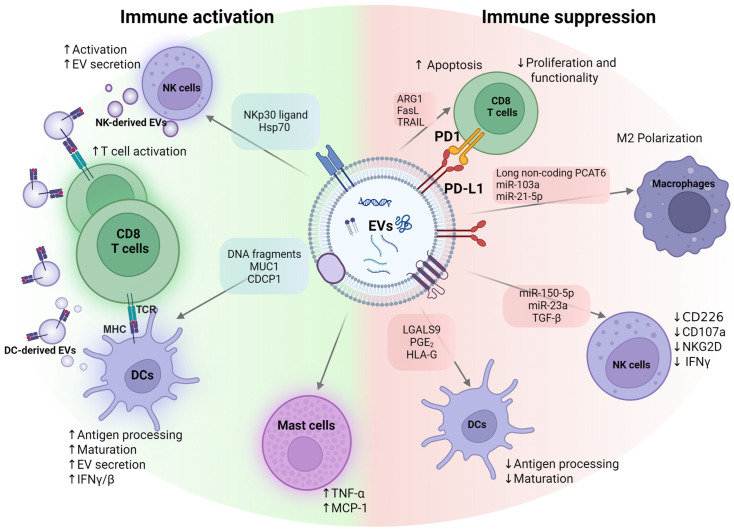
Immune modulation driven by EVs in LC. In LC, EVs are involved in both pro-tumoral and anti-tumoral processes. Indeed, on the one side, they inhibit the proper functionality of anti-tumor cell populations, such as CD8 T cells and NK cells, and at the same time foster the activity of pro-tumoral immune subsets, such as M2 macrophages. On the other side, EVs are directly involved in the activation of both innate and immune cells, thus boosting the recognition and elimination of cancer cells. Created in BioRender. Ghidotti, P. (2026) https://BioRender.com/trwfml1 (accessed on 19 June 2026).

Escaping the immune surveillance is a fundamental requirement for cancer cells to achieve primary tumor growth, but also dissemination and metastasis [126]. Cancer cells shed EVs that influence the immune system in two different ways [69]. Indeed, on the one hand, both surface proteins and the cargo of tumor EVs suppress the activity of different immune populations, such as NK cells and T cells. On the other hand, the presence of tumor-associated antigens (TAAs) on the surface of EVs facilitates the recognition and the exposure of these TAAs in antigen-presenting cells (APCs), thus starting an anti-tumor immune response [69,75]. The mechanisms of immune regulation mediated by EVs described in the following paragraphs are summarized in Figure 2.

#### 4.2.1. Immune Evasion

A well-described mechanism by which tumor EVs impair T cell functionality in LC and in many other tumor types is via the presence of the immune checkpoint inhibitory molecule PD-L1 on EVs. This inhibitory mechanism was first reported by Chen and colleagues in melanoma [127]. They showed that PD-L1^+^ EVs derived from melanoma cell lines suppressed the cytotoxic activity of CD8^+^ T cells in a co-culture experiment [127]. A following study of the same research group further shed light on how T cell-tumor EVs interaction happens in this context. In particular, PD-L1^+^ tumor EVs interact with T cells when ICAM-1 is also present on EV surface, thus allowing the interaction with LFA-1 on T cell surface [128]. After this first evidence in melanoma, many other studies highlighted that PD-L1^+^ EVs derived from other cancer types, such as LC [129], glioblastoma [130], and prostate cancer [131], showed comparable suppressive capability against CTLs, underlining a shared immunosuppressive mechanism. However, it is worth noting that all of these studies used EVs derived from tumor cell lines, and none of them provided evidence that this mechanism also works using circulating patient-derived EVs. In 2022, our group explored the possible immunosuppressive activity of circulating plasma EVs derived from metastatic NSCLC treated with immunotherapy [132]. Despite the presence of PD-L1 on patient-derived EVs, when those EVs were used in a functional experiment with T cells, we did not observe any modulation either in terms of T cell proliferation or in functionality [132]. Therefore, evidence remains to be provided in cancer patients that PD-L1^+^ EVs represent important players in T-cell suppressive anti-tumor activity.

In LC, PD-L1^+^ EVs not only target T cells but also influence macrophage polarization towards an M2 pro-tumoral phenotype [133]. Moreover, authors showed that once polarized, M2 macrophages support the proliferation and invasion of LC cells [133]. Polarization of TAMs is also mediated by the transfer of non-coding RNA by tumor EVs. The long non-coding RNA PCAT6 carried by LC cell-derived EVs, once internalized, inhibits miR-326 activity, allowing the signaling pathway mediated by KLF1, which eventually induces M2 polarization [134]. Also, miR-103a, enriched in EVs derived from LC cells exposed to hypoxic conditions, when transferred to TAMs, induces their polarization towards M2 phenotype by targeting the PTEN signaling pathway [135]. Moreover, hypoxia also increases the presence of miR-21-5p in EVs derived from mesenchymal stem cells, which again promotes M2 polarization that eventually supports LC cell survival by reducing their apoptotic rate [136].

Different studies also reported that hypoxia, by changing the cargo of LC-derived EVs, endows them with immunosuppressive properties against NK cells. Chang et al. reported that hypoxic LC-derived EVs reduce the expression of CD226 and functionality of NK cells by carrying miR-150-5p [137]. Instead, Berchem and colleagues reported an enrichment of miR-23a and TGF-β in hypoxic LC cell-derived EVs [138]. The transfer of both miR-23a and TGF-β via EVs to NK cells in vitro suppresses NK functionality by targeting CD107a and NKG2D, respectively [138]. Interestingly, the shuttling of TGF-β by tumor EVs represents a shared mechanism adopted by different cancer types to shut down the functionality and killing capabilities of NK cells, which are fundamental sentinels, in particular, to avoid cancer cell dissemination and metastasis [139,140,141].

DCs are professional APCs, and their proper functionality guarantees the subsequent activation of anti-tumor T cells [142]. However, DCs are also commonly impaired in their functionality after encountering tumor-derived EVs. Ning and colleagues reported that both Lewis lung carcinoma- and breast cancer-derived EVs, by affecting the differentiation and functionality of DCs, prevent the proper activity of CD4^+^IFNγ^+^ T cells [143]. DC inhibition is induced by tumor EVs also via several other mechanisms that involve the transfer of different miRNAs [144,145], and proteins such as LGALS9 [146], prostaglandin E2 [147], and non-classical human leukocyte antigen (e.g., HLA-G) [148].

#### 4.2.2. Immune Activation Mediated by Extracellular Vesicles

As anticipated, tumor-derived EVs can also elicit an immune response since they carry both TAAs and damage-associated molecular patterns (DAMPs) that can allow immune recognition and consequent anti-tumor immune response [69]. In LC, no direct evidence of anti-tumor immunity promoted by EVs has been described so far. However, as for common mechanisms of immune inhibition mediated by EVs that are shared in different cancer types, we are also convinced that findings described in other cancers can be relevant even in LC. Considering DCs, the dual function of tumor EVs as promoters of immune response is well documented. Tumor DNA fragments transported by EVs induce the activation of the cGAS/STING pathway in DCs with the consequent production of type I IFN that further sustains DC maturation and activation [149]. Another important mechanism by which tumor EVs sustain DC activation and functionality is the uptake of TAAs with their consequent processing and presentation via MHCs, as demonstrated for CDCP1^+^ and MUC1^+^ EVs [150,151]. In addition to presenting TAAs, DCs—when activated by tumor-EVs—also shed their own EVs, which, in hepatocellular carcinoma, showed the capability of priming CD8^+^ T cells and decreased the infiltration of Treg cells [152]. Moreover, Segura and colleagues suggested that the capacity of DC-EVs to induce T cell response is indeed mediated by the expression of MHC-II molecules and the adhesion molecule ICAM-1 [153].

Tumor-derived EVs can also induce the activation of NK cells by presenting them with the NKp30 ligand and Hsp70 [154,155]. On the other hand, NK-derived EVs are also endowed with potent anti-tumor properties. Lugini et al. reported that NK-EVs carry both Fas ligand and perforin with cytotoxic activity against different tested tumor cell lines [156]. Moreover, in addition to Fas ligand and perforin, Zhu and colleagues also reported the presence of TNF-α inside NK-derived EVs that both in vitro and in vivo strongly reduce melanoma cell proliferation [157]. Also, other immune populations are directly targeted by tumor-cell-derived EVs. Salamon et al. reported that EVs obtained from both NSCLC cell lines and surgical lung tissue specimens interact with mast cells, increasing both their migratory capacity and the production of cytokines such as TNF-α and MCP-1, which, in turn, may affect the behavior of other immune cells and cells of the TME [158]. Instead, glioblastoma-derived EVs have been reported as inhibitors of Treg cells, by affecting their proliferation and infiltration in an in vivo glioblastoma mouse model [159].

In conclusion, EVs are strongly implicated in the regulation of the immune system during cancer. This regulation can both foster immune suppression and tumor escape and promote tumor recognition and elimination. However, the relevance of these mechanisms in LC patients remains to be demonstrated. Indeed, all the experimental evidence gathered so far has been documented only in in vitro LC cell lines and in vivo with different mouse models. Understanding the real contribution of EVs in immune regulation in cancer in patients would be crucial to highlight processes that may influence the response and efficacy of immunotherapy, as well as identify new potential therapeutic targets to prevent immune inhibition or implement its activation.

## 5. EV-Based Therapeutics in Lung Cancer: From Preclinical Promise to Clinical Challenges

**Figure 3:**
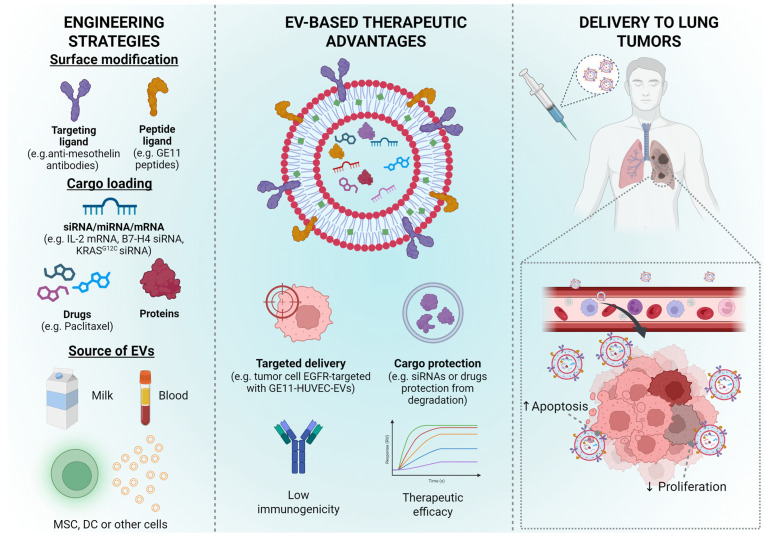
EVs as a therapeutic strategy in lung cancer. EVs represent an attractive platform from a therapeutic perspective. EVs of different sources can be functionalized with several strategies, such as the modification of surface molecules or the loading of specific nucleic acids and proteins. Functionalized EVs show different advantages as a therapeutic platform, considering their low immunogenicity, the stabilization of their cargo, and the efficient targeting of tumor cells in order to achieve high therapeutic efficacy. Once in the tumor, functionalized EVs aim to eradicate tumor cells by inducing apoptosis or by decreasing their proliferation rate, thus controlling tumor growth and possible metastatic dissemination. Created in BioRender. Fortunato, O. (2026) https://BioRender.com/oleaiy5 (accessed on 19 June 2026).

Recently, EVs have emerged as highly promising nanocarriers for drug delivery in cancer, thanks to their biocompatibility and low immunogenicity [160] (Figure 3).

In LC, extensive preclinical studies have demonstrated the potential of EVs to efficiently deliver their cargos, including chemotherapeutic agents, proteins, and nucleic acids, achieving improved targeting of the tumor microenvironment and reduced systemic toxicity compared to synthetic delivery systems [161]. These encouraging findings have positioned EV-based platforms as attractive candidates for next-generation precision oncology approaches [162]. However, the clinical translation of these systems in LC remains markedly limited [163]. To date, clinical trials have largely focused on the use of EVs as diagnostic or prognostic biomarkers in LC rather than as therapeutic vehicles for the boosting of anticancer immune responses [164]. Notably, studies such as the phase II trial by Besse and colleagues have demonstrated the safety and immunomodulatory potential of EV-based therapies, but have also highlighted their limited clinical efficacy and the absence of robust antigen-specific T-cell responses [165]. This study investigates DC-derived EVs (Dex) as a maintenance immunotherapy in patients with advanced NSCLC. This clinical approach builds on the concept that EVs released by DCs can stimulate antitumor immune responses, as underlined in Section 4.2.2. In this trial, Dex was administered to patients with stable or responding disease after chemotherapy. The treatment was found to be safe and well-tolerated, with no severe immune-related adverse events. Although no strong tumor regression was observed, the therapy showed immunomodulatory activity, particularly through the activation of NK cells rather than T-cell responses. The demonstration of safety in this study is particularly important given the challenges associated with immunotherapy toxicity. However, the study is limited by a relatively small sample size and the absence of a randomized control group, which restricts conclusions about efficacy. Clinical responses were modest, with no clear evidence of tumor regression, raising concerns about therapeutic potency. Furthermore, the lack of strong antigen-specific T-cell responses suggests suboptimal immunogenicity of the Dex formulation.

Concerning the pre-clinical studies, one of the most interesting and novel works investigates the potential role of EVs as a cancer vaccine. This study presents the development of an inhalable cancer immunotherapy based on extracellular vesicles loaded with IL-12 mRNA (IL-12-Exo) [166]. The authors engineered human embryonic kidney-derived EVs via electroporation to deliver IL-12 mRNA directly to the lung TME. In syngeneic and orthotopic mouse models of LC, inhaled IL-12-Exo achieved superior biodistribution compared to liposomes, while minimizing systemic toxicity. Functionally, IL-12-Exo induced strong local and systemic antitumor immune responses, primarily mediated by CD8^+^ T cells and dependent on IFNγ production. Importantly, localized delivery via inhalation avoided the severe systemic toxicity previously associated with recombinant IL-12 administration, likely by restricting cytokine exposure to tumor sites. Interestingly, the authors introduce an innovative, non-invasive delivery platform (inhalable exosomes) that effectively targets the lung, representing a clear advantage for LC therapy. The comparison with liposomal delivery strengthens the claim of improved biodistribution and safety. Additionally, the strategy addresses a well-known limitation of IL-12 therapy, which is the systemic toxicity, by localizing cytokine activity. Despite its strengths, the study is limited to mouse models, and the use of HEK-derived exosomes could pose scalability, safety, and regulatory challenges in humans that are not addressed. Electroporation-based mRNA loading may result in variable efficiency and exosome heterogeneity, but this has not been deeply characterized.

Recently, a study was published where investigators modified EVs to deliver therapeutic siRNA in LC cells [167]. They used a specific siRNAs against B7-H4, which is an immune-checkpoint molecule, encapsulated in nanoparticles with specific surface ligands that improve uptake by cancer cells. These EVs are able to reduce tumor growth and survival. Through in vitro experiments and in vivo models, the study evaluates how efficiently the engineered EVs deliver their cargo to lung tumor cells and their impact on tumor progression. The results suggest that these EVs can protect siRNA from degradation in circulation and facilitate its accumulation at the tumor site, leading to measurable therapeutic effects. However, as with all the studies above, the main limitation is the absence of validation in a human setting.

EVs from other sources were explored for drug delivery in cancer [168]. Interestingly, the use of milk-derived EVs as a novel nanocarrier for the oral delivery of paclitaxel, aiming to overcome the drug’s poor bioavailability and high toxicity associated with intravenous administration, was investigated by Agrawal et al. [169]. The authors demonstrate that paclitaxel-loaded EVs (ExoPAC) are stable in gastrointestinal conditions, provide sustained drug release, and significantly inhibit tumor growth in vivo compared to conventional administration. A major strength of the study is the innovative use of a natural, biocompatible delivery system that shows reduced systemic and immunological toxicity. However, the study is limited by its reliance on animal models, with no clinical validation in humans. Furthermore, drug-loading efficiency remains relatively low and large-scale reproducibility is not fully addressed. Concerning the large-scale production of EVs with modified cargos, an attempt was recently performed by using shock waves to encapsulate siRNAs against KRAS^G12C^ in milk-derived EVs to eliminate LC cells. This methodology increased the efficiency of encapsulation of siRNAs inside EVs compared to standard techniques, generating a compound that is able to reduce tumor growth in a xenograft mouse model [170].

To expand targeting applications, Gaurav et al. engineered GE11 peptide-modified EVs derived from human umbilical vein endothelial cells for vinorelbine delivery in LC [171]. This system achieved tumor-specific targeting via EGFR recognition, demonstrating potent antitumor effects and reduced off-target toxicity in xenograft murine models. The author used a postinsertion strategy that allows the modification of EVs after their isolation. However, even if it is demonstrated that the anticancer effects in mouse models, several efforts should be made to demonstrate safety and efficacy in humans to be used as a new therapeutic strategy in LC patients. Interestingly, Zheng and colleagues investigated the possibility of delivering EVs from CAR-T directly to the lungs by inhalation. These EVs were functionalized with anti-mesothelin antibodies to target Lewis lung carcinoma and loaded with paclitaxel. In orthotopic pulmonary mouse models, these CAR-EVs showed localization in the lungs with anti-tumoral effects by awakening T cell responses against LC cells [172].

The lack of advanced clinical studies employing EVs for direct therapeutic cargo delivery underscores several unresolved challenges, including large-scale production under GMP conditions, standardization of isolation and characterization methods, optimization of cargo loading efficiency, and off-target toxicity [173]. Furthermore, improving in vivo targeting efficiency remains a key challenge. Surface engineering of EVs through the incorporation of targeting ligands, peptides, antibodies or aptamers on the EV membrane aims to improve their targeting specificity toward the lung tumor microenvironment. However, these modifications involve complex manufacturing processes that may affect EV integrity, stability, and increase the risk of immunogenicity, limiting their clinical applicability. Furthermore, EVs can be modified for therapeutic purposes by loading with nucleic acids or proteins. Indeed, EVs can be engineered to carry miRNAs, siRNAs, proteins, enzymes, cytokines or genome-editing components that could modulate the phenotype of recipient cells. The advantages of using it as a delivery platform are the protection from enzymatic degradation in the circulation and the ease of cellular uptake. Nevertheless, challenges regarding loading efficiency, cargo stability, potential off-target effects and the scalability of manufacturing processes need to be addressed. Moreover, understanding the biodistribution, pharmacokinetics, and long-term immunological effects of EV-based therapies is essential for advancing their clinical application [174]. Together, these limitations reveal a significant gap between preclinical innovation and clinical implementation, emphasizing the need for further technological refinement and well-designed clinical trials to fully harness the therapeutic potential of EV-based drug delivery systems in lung cancer.

## 6. Future Perspectives and Clinical Translation

EVs have emerged as central regulators of immune communication within the lung TME, contributing to tumor progression, immune escape, and metastatic dissemination. At the same time, their intrinsic properties make them attractive candidates for biomarker discovery and innovative therapeutic drugs. Although preclinical evidence strongly supports their translational potential in NSCLC, technical and clinical challenges still hinder their widespread application. One of the major obstacles is the lack of standardized protocols for EV isolation and purification. Differences in EV isolation methods and analytical techniques often result in heterogeneous preparations, making comparisons among studies difficult and limiting reproducibility. Another critical issue is represented by large-scale production. While MSC-derived EVs have demonstrated promising therapeutic properties, obtaining high quantities of EVs remains technically demanding and costly. Future efforts should focus on the development of scalable manufacturing platforms, including bioreactor systems or alternative EV sources. In parallel, the development of EV mimetics may help overcome some of the limitations associated with natural EV yields and cargo-loading efficiency.

For the clinical applicability of EV in the management of NSCLC patients, particularly promising is the integration of EV-based therapies with standard therapies, such as the use of immune-checkpoint inhibitors. Since engineered EVs could modulate the TIME, the combination with immunotherapy could improve the efficacy of immune checkpoint inhibitors. Such combinatorial strategies could be important in overcoming resistance mechanisms that frequently limit the clinical benefit of current immunotherapeutic approaches in NSCLC.

Finally, the future of EV research in NSCLC may extend beyond therapeutic applications alone. The dual role of EVs as both biomarkers and therapeutic drugs supports the development of platforms capable of simultaneously monitoring disease progression and delivering targeted treatments. Although several EV-based clinical trials have already demonstrated favorable safety profiles, robust evidence of clinical efficacy is still limited. In conclusion, well-designed clinical studies, together with advances in manufacturing, standardization and regulatory frameworks, will be fundamental for the clinical applicability of EVs for lung cancer patients.

## Figures and Tables

**Table 1:** Shared immune regulatory mechanisms in cancer.

Immune Cell Population	Major Mechanism of Action	Reference
**CD8^+^ Cytotoxic T Cells (CTLs)**	- Recognize tumor antigens presented on MHC class I molecules - Promote tumor cell-induced apoptosis through the perforin/granzyme pathway - Activate death receptor signaling, mediated via FasL–Fas and TRAIL	[45]
**CD4^+^ Helper T Cells**	- Influence the activation state of CTLs - Th1 production of cytokines IFNγ, IL-2 and TNF-α with anti-tumor effects - Th9 release of IL-9 with pro-tumorigenic effects - Th2 release of IL-4 with pro-tumorigenic effects	[46]
**CD4^+^ Regulatory T Cells (Tregs)**	- Directly suppress CTLs through IL-10, TGF-β and IL-35 cytokine production - CTLA-4-mediated T cell inhibition	[47]
**Natural Killer (NK) Cells**	- Release of cytotoxic granules containing granzymes and perforins - Induce apoptosis through the expression of TRAIL receptor	[48]
**Macrophages**	- M1 phenotype endowed with anti-tumorigenic functions in contrast to M2, with pro-tumorigenic. - M2 produce IL-6, IL-8, and IL-10 - M2 recruit Tregs	[49]
**Dendritic Cells (DCs)**	- Present tumor-associated antigens to T cells to trigger antitumor immune responses - Secrete immunosuppressive factors, including cytokines (e.g., IL-8, IL-10, and TGF-β), growth factors (e.g., VEGF)	[50]
**B Cells**	- Present antigen to CD4^+^ T cells	[51]
**Myeloid-Derived Suppressor Cells (MDSCs)**	- Expression of negative immune checkpoint molecules such as PD-L1, VISTA or CD155. - Secretes a series of reactive oxygen and nitrogen species to damage T cell function	[52]
**Neutrophils**	- Promote tumor progression by inducing apoptosis in non-activated CTLs through mechanisms dependent on TNF-α and nitric oxide (NO) - Anti-tumoral effects by producing cytotoxic mediators or releasing TNF-related apoptosis-inducing ligands	[53,54]

**Table 2:** Components of EVs involved in the promotion of primary LC and LC metastasis.

EV’s Cargo	Origin	Mechanism of Action	Reference
EGFR protein	LC cell lines	Increase proliferation, migration, and invasion	[80]
c-Myc protein	LC cell lines	- Specific miRNA cargo sorting - Acquisition of aggressive traits in normal bronchial epithelial cells	[81]
EV-associated RNA	LC cell lines	Aggressive phenotype induction in normal bronchial epithelial cells	[98]
α-SMA	LC cell lines	- Inhibition of apoptosis - Increase proliferation	[99]
LRG1, miR-142-3p	LC cell lines	Angiogenesis induction via TGF-β inhibition	[100,101]
miR-23	LC cell lines	- Accumulation of HIF-1α → induction of angiogenesis - ZO-1 downmodulation → increase vascular permeability	[102]
EVs	LC cell lines	Angiogenesis promotion by inhibiting YAP signaling	[103]
α6β4 and α6β1	Human breast cancer cell lines	Binding to lung-resident fibroblast and epithelial cells to promote lung pre-metastatic niche formation	[104]
miR-92a	MDSCs from LC-bearing mice, serum-derived LC patients	ECM deposition by hepatic stellate cells	[105]
EVs	LC and melanoma cell lines mutated for p53	Less organized ECM production → tumor invasion in the LC pre-metastatic niche	[106]
EVs	Breast cancer cells	Neutrophil recruitment to the lung	[107]
EVs	Breast cancer cells	MDSCs accumulation, suppression of T cell proliferation and KN cytotoxicity → lung and liver metastatic promotion	[108]
EVs	Human breast cancer cell lines	Induce production of S100 in lung-resident fibroblasts → macrophages, DCs, and neutrophils recruitment to the lung	[104]
miR-3473b	Lewis lung carcinoma	Production of pro-inflammatory cytokines in fibroblasts → promotion of lung cancer metastatic invasion	[109]
miR-3157-3p	LC cell lines	- Vascular leakiness and angiogenesis - Lung metastatic nodule formation in vivo	[110]
miR-25-3p	Colorectal cancer cells	Vascular leakiness → enhances colorectal metastasis in the liver and lung of mice	[111]
miR-105	Metastatic breast cancer cells	ZO-1 reduces expression in endothelial cells → lung and brain metastatic induction	[112]

## Data Availability

No new data were created or analyzed in this study. Data sharing is not applicable to this article.

## Abbreviations

The following abbreviations are used in this manuscript:

APCs	Antigen-presenting cells
CSCs	Cancer stem cells
Th	CD4^+^ T helper cells
CTLs	Cytotoxic CD8^+^ T lymphocytes
CTLA4	Cytotoxic T-lymphocyte-associated protein 4
DAMPs	Damage-associated molecular patterns
Dex	Dendritic cell-derived extracellular vesicles
DCs	Dendritic cells
ECM	Extracellular matrix
EVs	Extracellular vesicles
GMP	Good manufacturing practices
ICBs	Immune checkpoint blockers
IFN-γ	Interferon-γ
LUAD	Lung Adenocarcinoma
LC	Lung Cancer
LUSC	Lung Squamous Cell Carcinoma
MHC	Major histocompatibility complex molecules
miRNAs	MicroRNAs
M-MDSCs	Monocytic myeloid-derived suppressor cells
MDSCs	Myeloid-derived suppressor cells
NK cells	Natural Killer cells
NO	Nitric oxide
NSCLC	Non-small cell lung cancer
OS	Overall survival
PMN-MDSCs	polymorphonuclear myeloid-derived suppressor cells
PMN	Pre-metastatic niche
PD-1	Programmed cell death protein 1
PD-L1	Programmed death-ligand 1
PFS	Progression-free survival
Treg	Regulatory T cells
SCLC	Small-cell lung cancer
TIME	Tumor immune microenvironment
TME	Tumor microenvironment
TMB	Tumor mutational burden
TRAIL	Tumor necrosis factor-related apoptosis-inducing ligand
TAAs	Tumor-associated antigens
TAMs	Tumor-associated macrophages
VISTA	V-domain Ig suppressor of T-cell activation
WHO	World Health Organization
